# Unveiling the Enigma of Matter under Extreme Conditions: From Planetary Cores to Functional Materials

**DOI:** 10.1038/s41598-023-45240-x

**Published:** 2023-10-26

**Authors:** Yang Song, Wei Luo, Yuejian Wang, Changqing Jin

**Affiliations:** 1 Department of Chemistry, The University of Western Ontario, London, ON N6A 5B7 Canada; 2 Department of Physics and Astronomy, Uppsala University, Uppsala, Sweden; 3 Department of Physics, Oakland University, Rochester, MI 48309 USA; 4 Institute of Physics, Chinese Academy of Sciences, Beijing, 100190 China

**Keywords:** Materials science, Planetary science

## Abstract

Pressure, as one of the fundamental thermodynamic parameters, can profoundly change the interatomic distances, electronic interactions, chemical bonding and crystal structures, leading to exotic structures and properties of materials. High-pressure techniques have significantly impacted disciplines like physics, chemistry, geology, and life science, creating new materials, advancing knowledge of Earth's interior, and influencing pharmaceutical development. This editorial reviews the latest research published in this Collection, highlighting the potential of high-pressure studies to further our understanding of materials’ behavior under extreme conditions.

Since Percy Bridgeman was awarded Nobel Prize in physics 1946 "for the invention of an apparatus to produce extremely high pressures, and for the discoveries he made therewith in the field of high pressure physics", studies of matter under extreme conditions such as under high pressures has expanded exponentially especially in the recent a few decades. The continuous development of high-pressure techniques and instruments, such diamond anvil cells, in situ optical and electrical probes, and especially advanced synchrotron sources, is pushing these frontiers even further by revealing unprecedented high-pressure phenomena at microscopic level. Simultaneously, simulations based on advanced computational theories and more recently artificial intelligence not only aid the understanding of these complex high-pressure phenomena, but also provide guidance for future development of novel materials using high pressure as a unique tool.

High-pressure science has become a cornerstone in a wide range of interdisciplinary frontiers, leading to numerous groundbreaking discoveries. In condensed matter physics, high-pressure studies have led to the development of new materials with unusual properties, such as superconducting hydrides or even near-room temperature superconductors that are being pursued aggressively^1,2^. In chemistry and materials science, the synthesis of novel functional materials such as superhard materials, non-conventional non-stoichiometry compounds, which are stable only under high-pressure conditions, has been realized^3,4^. In earth and planetary sciences, high-pressure experiments have been critical for our understanding of Earth’s deep interior, providing insights into seismic activity, plate tectonics, and the formation of mineral deposits^5–8^. In life sciences, high-pressure studies have contributed to advancements in areas such as enzyme kinetics, protein structure, and pharmaceuticals^9^. This Collection of original research articles represents selected examples in the above sub-disciplines that reflect the rich diversity and potential of high-pressure materials research.

One of the most prominent applications of high pressure is the study of geological and planetary processes and materials. Planetary core science is experiencing an epoch of transformation, as novel insights about the cores of Earth, Mercury, Venus, and the Moon are emerging. The studies on Fe–Ni–Si and Fe–Si–S alloys, as well as their resistivity, carry profound implications for understanding the thermal convection and magnetic fields within these celestial bodies^10,11^. These explorations can alter our understanding of planetary evolution and perhaps even illuminate the mysteries of our own planet’s dynamo. Expanding into the realm of geochemistry, the exploration of water dissolution in albite melt at high pressures and temperatures offers new insights into the behavior of hydrous silicate melts under extreme conditions^12^. Such findings have broad implications for understanding geological phenomena such as volcanic eruptions and plate tectonics.

Utilization of high pressure as a driving force to develop functional materials for clean energy applications represents another vibrant area of high-pressure materials research. New functional materials for batteries, photovoltaics, light emitting diodes (LED), and hydrogen storage devices are of fundamental importance for conversion, harvest, and storage of clean energy. Pressure has been shown to be an effective tool to tune the structures and properties and thus the performance of these functional materials^13^; in particular, advancements in high-pressure optoelectronics offer new pathways for space photovoltaics. The properties of the Cd_0.25_Zn_0.75_Se alloy under high pressure show the potential to broaden the spectrum of light absorption, thereby potentially boosting the efficiency of space-based solar cells^14^. For another example, the exploration of structure-dynamic-disorder relationships in metal halide perovskites (MHPs) under pressure further demonstrates the transformative power of pressure^15^. These discoveries deepen our understanding of MHPs, which are crucial to the optoelectronic devices like solar cells and LEDs.

Furthermore, development of energy storage materials using high-pressure techniques offers a promising alternative to conventional approaches. In this regard, significant findings in terms of new high-pressure polymorphs in lithium-palladium–hydrogen systems are reported^16^. In particular, the role of hydrogen is found to have important implications in altering the structures of these compounds. These studies provide valuable insights into the behavior of these systems under high pressure and temperature, offering vital inputs for energy storage and conversion technologies.

The exploration of high-pressure systems extends to ionic liquids, with significant findings on the influence of bulky anions on liquid–liquid phase transitions^17^. It is reported that identification of strong van der Waals interactions between alkyl chains of cations and anions in certain ionic liquids, as well as the observation that these interactions and the structure of the anions significantly influence the pressure sensitivity of the glass transition temperature. This work provides unique insights into the complex dynamics of ionic liquids under varied conditions, potentially leading to new technologies and methodologies.

Finally, in the pharmaceutical realm, high-pressure studies on ticagrelor, an active pharmaceutical ingredient, reveal the molecule's sensitivity to pressure^18^. Specifically, the study finds that the hydrogen-bonding pattern in ticagrelor remains unchanged under compression, which influences its thermal properties, and that the activation entropy slightly increases with compression, suggesting intermolecular cooperativity. This understanding can impact how we develop and manufacture drugs, potentially leading to improved stability and efficacy.

Overall, as demonstrated by the articles in this Collection, high-pressure materials science is a field of exploration and discovery, with the potential to not only advance our understanding of the world around us but also drive the development of new technologies and solutions for the future. We guest editors are aware that this Collection only encompasses a limited range of the exciting, expansive, and rapidly advancing field of high-pressure materials research. Finally, we express our gratitude to all authors contributing to this special Collection of “Materials under High Pressure” and hope the community finds their work interesting and enjoyable.
